# High-resolution genotyping of Lymphogranuloma Venereum (LGV) strains of *Chlamydia trachomatis* in London using multi-locus VNTR analysis-*ompA* genotyping (MLVA-*ompA*)

**DOI:** 10.1371/journal.pone.0254233

**Published:** 2021-07-08

**Authors:** Chloe Manning, Colette O’Neill, Ian N. Clarke, Monica Rebec, Penelope R. Cliff, Peter Marsh

**Affiliations:** 1 Department of Molecular Microbiology, Division of Clinical and Experimental Sciences, Faculty of Medicine, University of Southampton, Southampton, United Kingdom; 2 Department of Microbiology, Imperial College Healthcare NHS Trust, Charing Cross Hospital, London, United Kingdom; 3 Department of Infection Sciences, St Thomas’ Hospital, London, United Kingdom; 4 Public Health England, Porton Down, Salisbury, United Kingdom; GGD Amsterdam, NETHERLANDS

## Abstract

**Background:**

Lymphogranuloma venereum (LGV) is caused by *Chlamydia trachomatis* strains with *ompA* genotypes L1 to L3. An LGV epidemic associated with the L2b genotype has emerged in the past few decades amongst men who have sex with men (MSM). *C*. *trachomatis* genotypes can be discriminated by outer membrane protein A gene (*ompA*) sequencing, however this method has limited resolution. This study employed a high-resolution genotyping method, namely, multi-locus tandem repeat (VNTR) analysis with *ompA* sequencing (MLVA-*ompA*), to assess the distribution of LGV MLVA-*ompA* genotypes amongst individuals attending genitourinary medicine (GUM) clinics in London.

**Methods:**

Clinical specimens were collected from individuals attending eight London-based GUM clinics. Specimens that tested positive for *C*. *trachomatis* by commercial nucleic acid amplification test (NAAT) were confirmed as LGV by *pmpH* real-time PCR. LGV-positive DNA extracts were subsequently genotyped using MLVA-*ompA*.

**Results:**

Two hundred and thirty DNA extracts were confirmed as LGV, and 162 (70%) yielded complete MLVA-*ompA* genotypes. Six LGV MLVA-*ompA* genotypes were identified: 1.9.2b-L2, 1.9.3b-L2b, 1.9.2b-L2b, 1.9.2b-L2b/D, 1.4a.2b-L2b, and 5.9.2b-L1. The following LGV *ompA* genotypes were identified (in descending order of abundance): L2, L2b, L2b/D, and L1. Eight *ompA* sequences with the hybrid L2b/D profile were detected. The hybrid sequence was identical to the *ompA* of a recombinant L2b/D strain detected in Portugal in 2017.

**Conclusions:**

The L2 *ompA* genotype was found to predominate in the London study population. The study detected an unusual hybrid L2b/D *ompA* profile that was previously reported in Portugal. We recommend further monitoring and surveillance of LGV strains within the UK population.

## Introduction

Lymphogranuloma Venereum (LGV) is caused by *Chlamydia trachomatis ompA* genotypes L1, L2 and L3 [1]. In 2003, an outbreak of LGV was reported in a sexual network of men who have sex with men (MSM) presenting with symptoms of proctitis in Rotterdam [2]. Sequencing of the *ompA* gene demonstrated that the initial outbreak was caused by a new genetic variant designated L2b [3]. This variant has subsequently been implicated in LGV outbreaks worldwide [4–10]. The United Kingdom has the highest number of confirmed LGV cases in Europe, with a total of 6,752 UK LGV diagnoses made between 2003 and 2018 [11, 12]. A report showed that 617/919 (67%) of UK LGV diagnoses were made in London [11].

Whole genome sequencing has proven informative for elucidating the evolutionary history and diversity of *C*. *trachomatis* [13, 14], however the high resolution provided by this method is not always necessary [15]. For decades, sequence analysis of the *ompA* gene was used to differentiate *C*. *trachomatis* strains [16]. However, the *ompA* gene is not always an accurate epidemiological marker when used on its own, with *ompA* shown to be a recombination hotspot in the genome [13]. *OmpA* genotyping has been largely superseded by more discriminatory genotyping systems including multi-locus sequence typing (MLST) [15, 17], and multi-locus variable number tandem repeat (VNTR) analysis with *ompA* genotyping (MLVA-*ompA*) [18].

MLVA-*ompA* targets variation in the number of repeating mononucleotides at three VNTR loci dispersed throughout the chlamydial genome (i.e. CT1335, CT1299, and CT1291), coupled with sequencing of the *ompA* gene [18]. The MLVA-*ompA* system has been successfully applied to genotype *C*. *trachomatis* strains globally [19–23]. MLVA-*ompA* has a high degree of resolution which is essential for isolate discrimination, with earlier studies reporting a discriminatory index between 0.94 and 0.99 [18, 24], as measured by Simpson’s Index of Diversity [25]. Whilst MLVA-*ompA* has not previously been applied on a large-scale to genotype LGV strains in the United Kingdom; LGV clinical samples have been genotyped successfully using the system in Brighton (n = 11) [19] and Southampton (n = 1) [24]. The study in Brighton identified nine distinct LGV MLVA-*ompA* genotypes, with seven genotypes detected within isolates assigned an *ompA* genotype L2b [19]. The genotypic diversity exhibited within LGV strains in Brighton raised questions about whether a similar extent of genotypic diversity might exist within LGV strains circulating in other UK cities. Given the high prevalence of LGV in London and its substantial MSM population [11], it was decided to apply the MLVA-*ompA* system to genotype LGV clinical DNA extracts from this region. The aim of this study was to assess the distribution of LGV MLVA-*ompA* genotypes from clinical specimens sourced from individuals attending eight London-based GUM clinics.

## Methods

### Clinical specimens

Clinical specimens including rectal swabs, throat swabs, urine and “pooled 3-in-1” specimens (a rectal swab and pharyngeal swab in urine, from a single patient) were collected from patients attending eight London-based GUM clinics. Specimens collected from 56 Dean Street, John Hunter Clinic, 10 Hammersmith Broadway, and Jefferiss Wing were tested for *C*. *trachomatis* at North West London Partnership (NWLP), hosted by Imperial College Healthcare NHS Trust; and specimens collected from Burrell Street Clinic, Streatham Hill Clinic, Walworth Road Clinic, and Harrison Wing were tested for *C*. *trachomatis* at Viapath, a private-sector diagnostic laboratory based at St Thomas’ Hospital.

At Viapath, the Aptima CT/NG Combo 2 Assay (Hologic^®^, US) was used for *C*. *trachomatis* detection. NAAT-positive specimens from MSM were reflex tested for LGV at Viapath. Nucleic acids were extracted using the Complex 200 Protocol for the QIAsymphony DSP Virus/Pathogen kit (QIAGEN, US), followed by an in-house triplex LGV PCR assay targeting the *pmpH* gene located on the *C*. *trachomatis* chromosome; in addition to an 88-base pair region of the *C*. *trachomatis* cryptic plasmid; and the human *RNase P* gene as an internal control [26].

For the majority of clinics that send specimens to NWLP for *C*. *trachomatis* testing, there is a reflex to LGV test all rectal NAAT-positive specimens. At NWLP, the BD ProbeTec^™^ CT/GC Amplified DNA Assay (Becton-Dickinson, US) was used for *C*. *trachomatis* detection. Purified nucleic acids were isolated from each specimen using the MagNA Pure Compact Nucleic Acid Isolation Kit I (Roche Life Science, UK) before application of the triplex LGV PCR assay (as described for Viapath) [26].

For this study, DNA extracts that were confirmed as LGV at NWLP and Viapath were selected consecutively with no bias for positivity strength. DNA extracts included in this study were collected from patients between February 2018 and June 2019. DNA extracts were transported on dry ice to the University of Southampton Molecular Microbiology Group for genotyping. All patient-identifiable information was removed from each DNA extract prior to dispatch to Southampton. Extracts were subsequently stored at -20°C.

### PCR amplification of VNTR and *ompA* sequences

VNTR and *ompA* sequences were amplified from the DNA extracts using PCR according to Wang *et al* [22]. For *ompA*, a fragment of this gene (ca 1,000bp) was amplified using primers PCTM3 and NR1 [27], whilst the three VNTR regions were amplified using primers described by Pedersen *et al* [18]. Extracts that did not produce VNTR amplicons using these primers were amplified using primers CT1335F* and CT1335R*, CT1299F* and CT1299R*, and CT1291F* and CT1291R* [28] (S1 Table). The forward primers annealed upstream of the original VNTR amplicon sequences, and the reverse primers downstream, so that the original amplicon sequences were encompassed by the alternative primers. PCR reactions were carried out in 20 μL volumes consisting of: 10 μL Phusion Flash High-Fidelity PCR Master Mix (Thermo Scientific^™^, UK), 0.5μM of the forward and reverse primers (Eurogentec, Belgium), and 1 μL of DNA. PCR products were loaded onto 2% (w/v) agarose gels for the purpose of checking amplicon size and quality. The amplicons were subsequently purified using the Wizard SV Gel and PCR Clean-Up System (Promega, UK) for sequencing. PCR amplicons were commercially sequenced at Source Bioscience (Cambridge, UK).

### DNA sequence analysis of MLVA-*ompA* markers

Alphabetical *ompA* genotypes were assigned to each extract via *ompA* sequence comparison to the NCBI database using Basic Local Alignment Search Tool (BLAST) [22]. VNTR sequences were compared to those described in Pedersen *et al* [18], Wang *et al* [22], Labiran *et al* [24], and Satoh *et al* [21]; and a single-digit number assigned at each VNTR locus based on the number of repeating mononucleotides [22]. The final MLVA-*ompA* genotype was designated by a three-digit code in the order: CT1335, CT1299, and CT1291, followed by the alphabetical *ompA* genotype (e.g. 3.9.3-G).

### Bioinformatics

To confirm the VNTR profile of the Portuguese strain (strain Ct_L2b/D_PT05; European Nucleotide Archive (ENA) accession number CAAKND010000000) [29] *in silico*,. fastq files were downloaded and converted to .fasta format using the conversion tool on the public server at usegalaxy.org. The resulting .fasta files were opened using the BioEdit (version 7.0.5.3) alignment software, and the VNTRs were located by inputting the VNTR primer sequences into the search tool.

### Ethics

This study was approved by the East of Scotland Research Ethics Committee (REC reference 19/ES/0012). All DNA extracts were received anonymised and unlinked. No patient clinical data, including age, sex, risk behaviour and clinical symptoms, was collected for this study. The requirement for informed consent was waived by the ethics committee.

## Results

### Clinical DNA extracts

A total of 230 DNA extracts that were confirmed as LGV using the *pmpH* real-time PCR-based assay were obtained for this study. These included 180 DNA extracts from NWLP and 50 DNA extracts from Viapath. Extracts from NWLP were from rectal swabs, and extracts from Viapath were from 23 pooled “3-in-1” specimens, 24 rectal swabs, 1 throat swab and 2 urines.

### *OmpA* genotypes identified in this study

One hundred and seventy three extracts (75.2%) were assigned an *ompA* genotype in this study (S2 Table). Of these, 164 extracts (94.8%) were assigned an LGV *ompA* genotype, and 9 were assigned non-LGV *ompA* genotypes. The most prevalent LGV *ompA* genotype identified in the study was L2 (n = 81, 49.3%), followed by L2b (n = 72, 43.9%). The non-LGV *ompA* genotypes identified were genotypes E (n = 3), G (n = 5), and J (n = 1).

### MLVA-*ompA* genotypes identified in this study

Sequence data were obtained for all four loci for 162/230 (70.4%) of the extracts (S2 Table). Of these, 159 (98.1%) were assigned LGV MLVA-*ompA* genotypes, and the remaining 3 extracts were assigned 3.9.3-G. Six distinct LGV MLVA-*ompA* genotypes were identified in this study (Table 1). The most prevalent LGV MLVA-*ompA* genotypes were 1.9.2b-L2 (n = 78, 49.1%), 1.9.2b-L2b (n = 53, 33.3%), and 1.9.3b-L2b (n = 16, 10.1%). Also detected were 5.9.2b-L1 (n = 3, 1.9%), 1.4a.2b-L2b (n = 1, 0.6%), and 1.9.2b-L2b/D (n = 8, 5.0%).

**Table 1:** Complete LGV MLVA-*ompA* genotypes identified in this study (n = 159).

*ompA*	MLVA†	n (% of 159 extracts)
L1	5.9.2b	3 (1.9)
L2	1.9.2b	78 (49.1)
L2b	1.4a.2b	1 (0.6)
	1.9.2b	53 (33.3)
	1.9.3b	16 (10.1)
L2b/D	1.9.2b	8 (5.0)

^†^ MLVA genotype was designated by the 3 VNTR loci in the order: CT1335; CT1299; and CT1291.

Extracts with partial MLVA-*ompA* profiles were excluded from this table.

#### VNTR sequence variants identified in the study

The VNTR variant code, CT1291 type 3b (AAAATAGTCTA-**9C**-TATTG), was identified in 20 extracts in this study. Sixteen extracts with the 3b variant code could be assigned an *ompA* genotype, of which all were *ompA* genotype L2b (S2 and S3 Tables). The complete MLVA-*ompA* genotype of these sixteen extracts was 1.9.3b-L2b. The CT1291 type 3b was previously identified by Satoh *et al* [21] and assigned to the reference strains L1/440/Bu and L2/434/Bu.

VNTR variants identified by Pedersen *et al* [18] and Wang *et al* [22] were detected in this study (S3 Table). The extract assigned a CT1299 type 4a in this study was the 4a variant (TTTTTATTCT-**10C-**T3C-ATCAAA) first identified in Wang *et al* [22]. All extracts assigned a CT1291 type 2b in this study were the 2b variant (AAAATAGTCTA-**8C**-TATTG) initially identified in Wang *et al* [22] (S3 Table).

In our study, CT1299 VNTR type 9 (TTTTTATTCT-3C2T-**6C**-ATCAAA) was assigned to 161/164 (98.2%) extracts with LGV *ompA* genotypes (S3 Table).

#### Detection of a hybrid L2b/D *ompA* genotype

We identified eight *ompA* sequences with an L2b/D hybrid profile (S1 Fig), i.e. whilst the first 365bp (numbers given relative to L2b/UCH-1/proctitis) of each sequence was identical to *ompA* L2 and L2b reference sequences (L2/434/Bu and L2b/UCH-1/proctitis); the region spanning 366bp-1,023bp revealed an *ompA* genotype D profile matching the reference strain D/UW-3/CX (Genbank accession no. NC_000117.1). Nucleotide BLAST of the eight sequences indicated 100% sequence identity to the *ompA* sequence of a novel hybrid L2b/D strain identified in Portugal [29] (Genbank accession no. MN094864.1). The eight hybrid sequences were designated L2b/D, to distinguish them from extracts with an *ompA* sequence matching L2b/UCH-1/proctitis.

All extracts assigned the L2b/D *ompA* genotype could be assigned a full MLVA-*ompA* genotype, all eight of which were 1.9.2b-L2b/D (S2 Table). We confirmed the VNTR profile of the Portuguese strain (ENA accession number CAAKND010000000) *in silico*: GAAAAAG-**9T8A**-GCTTTTGT at CT1335 (CT1335 type 1), TTTTTATTCT-3C2T-**6C**-ATCAAA at CT1299 (CT1299 type 9), and AAAATAGTCTA-**8C**-TATTG at CT1291 (CT1291 type 2b), corresponding to the same MLVA-*ompA* genotype of 1.9.2b-L2b/D as identified in this study.

## Discussion

This study represents the first MLVA-*ompA* genotyping survey of LGV strains of *C*. *trachomatis* in a London population. Amongst the extracts that could be assigned a type at all four loci, we identified six distinct LGV MLVA-*ompA* genotypes: 5.9.2b-L1, 1.9.2b-L2, 1.9.2b-L2b, 1.9.3b-L2b, 1.4a.2b-L2b, and 1.9.2b-L2b/D.

In accordance with a previous study [30], we found that the L2b *ompA* variant was not the most common in the pooled data set; the L2 *ompA* sequence predominated. However, in the previous study this varied by country, and the L2 *ompA* sequence predominated in Austria and Croatia, whilst the L2b *ompA* sequence predominated in the UK. Whilst the predominance of the L2 *ompA* genotype has been documented in France [31], Sweden [5], Spain [32], and Austria and Croatia [30], this is the first report of its predominance within the United Kingdom. A retrospective study of LGV *C*. *trachomatis* strains collected in France between 2010 and 2015 by Peuchant *et al*. [31] demonstrated that the proportion of LGV cases caused by L2b declined after 2012, whilst the proportion caused by *ompA* genotype L2 increased from 2012 onwards, which supports our findings. Our study showed that of those L2 extracts with a complete MLVA-*ompA* genotype (78/81, 96.3%), 100% had the MLVA-*ompA* genotype 1.9.2b-L2. This VNTR profile, 1.9.2b, was found to be shared amongst extracts designated L2, L2b and L2b/D in this study. These data show that at least three LGV *ompA* genotypes with the same VNTR profile are co-circulating within the London population. This is likely due to recombination within the *ompA* gene, given that *ompA* is known to be a recombination hotspot within the chlamydial genome [13, 33].

We noted the most diversity in MLVA-*ompA* genotypes within extracts designated *ompA* genotype L2b, with VNTR profiles 1.9.2b, 1.9.3b, and 1.4a.2b assigned. Diversity in L2b genotypes has previously been reported in Brighton [19]. Interestingly, whilst 1.9.2b-L2b and 1.9.3b-L2b comprised 53/159 (33.3%) and 16/159 (10.1%) respectively of all extracts assigned a complete LGV MLVA-*ompA* genotype in this study (Table 1); the Brighton study reported only three LGV cases with 1.9.2b-L2b and none with 1.9.3b-L2b. Further, the Brighton study identified eight LGV MLVA-*ompA* genotypes that were not detected in our London study population. These regional differences in MLVA-*ompA* genotypes between Brighton and London are likely the result of distinct dissemination patterns within each population; however, given that the Brighton study took place between 2011 and 2013, and the specimens for this London study were collected between 2018 and 2019; these differences could represent a temporal shift in genotypes.

We detected a hybrid L2b/D *ompA* sequence in DNA extracts from Viapath (n = 1) and NWLP (n = 7). All were assigned the MLVA-*ompA* genotype 1.9.2b-L2b/D. The hybrid *ompA* sequence was identical to the *ompA* of a recombinant L2b/D strain detected in Portugal [29] (S1 Fig). As of 2019, a total of 25 cases of the recombinant L2b/D strain have been reported in Portugal. Our study is the first report of the hybrid L2b/D *ompA* sequence in the United Kingdom. We demonstrated by *in silico* means that the Portuguese L2b/D strain possessed the 1.9.2b-L2b/D MLVA-*ompA* genotype. This result confirmed that the Portuguese L2b/D strain and the eight extracts assigned L2b/D from our London study population shared the same MLVA-*ompA* genotype. Whole genome sequencing of the Portuguese strain performed by Borges *et al*. [29] revealed that the strain resulted from the transfer of a 4.2kbp fragment from a *C*. *trachomatis* D strain to an L2b strain. The recombinant fragment comprised 75% of the *ompA* gene encoding the major outer membrane protein (MOMP), and four genes downstream of *ompA* each with functional roles in protein synthesis. This widespread genetic recombination, particularly within the MOMP epitope region that is responsible for influencing the ability of *C*. *trachomatis* strains to interact with the host immune response [34, 35], may have implications for the transmission and pathogenic capability of the hybrid strain [36, 37]. A limitation of our study was that we did not collect clinical data relating to patient symptoms, and as a result, we are unable to comment on the clinical presentation of patients with the L2b/D *ompA* sequence in the London study population. However it was noted by Borges *et al*. [29] that all of the individuals infected with the hybrid L2b/D strain presented with similar symptoms and clinical features (i.e. rectal pain, anal discharge and rectal bleeding), that are consistent with a typical LGV infection [1]. Of note, many of the individuals infected with the L2b/D strain in Portugal were involved in international sexual networks, which would explain how the variant likely reached our study population.

We detected the CT1291 variant code, type 3b (AAAATAGTCTA-**9C**-TATTG) (S3 Table) in 20 extracts. Sixteen of these extracts could be assigned a complete MLVA-*ompA* genotype, of which all were 1.9.3b-L2b. Prior to this study, CT1291 type 3b was detected by Satoh *et al*. in the L1/440/Bu and L2/434/Bu reference strains [21], and these were assigned the MLVA-*ompA* genotypes 5.9.3b-L1 and 5.9.3b-L2. The Satoh *et al*. study did not include any L2b isolates. The CT1291 type 3b was not detected in any of the 44 clinical isolates also MLVA-*ompA* genotyped by Satoh *et al*. In this study, we also detected VNTR variant codes first identified in Pedersen *et al* [18] and Wang *et al* [22], including CT1299 type 4a (TTTTTATTCT-**10C**-T3C-ATCAAA), CT1299 type 9 (TTTTTATTCT-3C2T-**6C**-ATCAAA), and CT1291 type 2b (AAAATAGTCTA-**8C**-TATTG).

The MLVA-*ompA* genotyping method had limited resolution when applied to our London study population, with three LGV MLVA-*ompA* genotypes (1.9.2b-L2, 1.9.3b-L2b, and 1.9.2b-L2b) comprising 92.5% of extracts assigned complete MLVA-*ompA* genotypes in the study. As a result, we did not reach a Simpson’s index of diversity of 0.95, the index value for a genotyping system to be considered to have more or less “ideal” resolution [38].

We detected the non-LGV *ompA* genotypes E (n = 3), G (n = 5) and J (n = 1) in this study. Genotypes G and J have commonly been found in the rectum of MSM [39, 40], and co-infections of LGV and urogenital genotype E infection have been reported previously [8]. However, extracts that were genotyped in this study were those that had given a positive result in the LGV biovar assay at NWLP and Viapath; that is, only extracts with the 36-bp deletion within the *pmpH* gene that is characteristic of LGV strains. Given the low prevalence of these non-LGV *ompA* genotypes in our study (9/173, 5.2%, of those with an assigned *ompA* genotype), there are a few possible explanations for their detection. Firstly, the nine extracts could have been false positives of the LGV biovar assay. The likelihood of this is slight—the assay has demonstrated excellent diagnostic performance in differentiating LGV and non-LGV infections in previous studies [41]. It is more likely that the individuals with a non-LGV *ompA* genotype in this study were infected with both an LGV strain and a non-LGV strain of *C*. *trachomatis*. Another possible explanation is that mixed infection could have resulted in *pmpH* variants caused by genetic exchange between LGV and genotype G, J or E *C*. *trachomatis* strains [8]. Given that the evolution of *C*. *trachomatis* is mainly driven by recombination [42], and many studies have reported co-infections with LGV strains and non-LGV *C*. *trachomatis* strains with invasive and non-invasive urogenital *ompA* genotypes [8, 42, 43], this explanation is still plausible. Mixed infections can help to facilitate the selection of new recombinants, such as the hybrid Portuguese strain [29], and the L2c strain described by Somboona *et al* [44], that were both caused by unique recombination events between L2b (and L2, respectively) and D genotypes.

## Conclusions

In conclusion, we have demonstrated that the predominant *ompA* genotype within our London study population is the L2 *ompA* genotype, and not the L2b *ompA* genotype that has been reported in UK populations since 2005 [45]. We provide the first UK report of a hybrid L2b/D *ompA* profile previously detected in Portugal. These findings highlight the ever-changing nature of the LGV epidemic, and we urge for attentive LGV surveillance strategies to continue.

## Supporting information

S1 Table Primer sequences for PCR of MLVA-*ompA* markers. (DOCX)

S2 Table Distribution of MLVA-*ompA* genotypes from clinical DNA extracts sourced from individuals attending eight London-based GUM clinics (n = 230). (XLSX)

S3 Table VNTR sequence analysis of extracts assigned LGV *ompA* genotypes (n = 164). VNTR type codes of extracts that were assigned non-LGV *ompA* genotypes (n = 9) or that could not be assigned an *ompA* genotype (n = 57), were excluded from this table.
(DOCX)

S1 Fig Alignment of partial *ompA* gene from extracts possessing a hybrid L2b/D-Da *ompA* profile (n = 8). The *ompA* sequence of L2b/UCH-1/proctitis (Genbank accession no. AM884177.1) was used as a reference. Nucleotide numbers are given according to L2b/UCH-1/proctitis. L2b/D refers to the *ompA* sequence of the Portuguese L2b/D strain (Genbank accession no. MN094864.1). The *ompA* sequences of L2/434/Bu (Genbank accession no. AM884176.1) and D/UW-3 (Genbank accession no. NC_000117.1) were included in the alignment. Coloured dots indicate bases that matched L2b/UCH-1/proctitis.
(DOCX)
