# Supplementary material for: High-resolution genotyping of Lymphogranuloma Venereum (LGV) strains of Chlamydia trachomatis in London using multi-locus VNTR analysis-ompA genotyping (MLVA-ompA)
Source: PLoS One. 2021 Jul 8;16(7):e0254233. doi: 10.1371/journal.pone.0254233 (PMC8266103; doi:10.1371/journal.pone.0254233)
Supplement: S1 Table — (DOCX) [file pone.0254233.s001.docx]

**S1 Table** **Primer sequences for PCR of MLVA-*ompA* markers**

|  |  |  |  |  |  |  |
| --- | --- | --- | --- | --- | --- | --- |
|  | **Primer Name** | **Primer sequence (5’- 3’)** | **Amplicon size^a^ (bp)** | **Nucleotide position^a^** | **Reference** |  |
|  | CT1335F | TCATAAAAGTTAAATGAAGAGGGACT | 153 | 737,225–737,250 | (18) |  |
|  | CT1335R | TAATCTTGGCTGGGGATTCA |  | 737,377–737,358 |  |  |
|  | CT1299F | TTGTGTAAAGAGGGTCTATCTCCA | 188 | 291,758–291,781 |  |  |
|  | CT1299R | AAGTCCACGTTGTCATTGTACG |  | 291,945–291,924 |  |  |
|  | CT1291F | GCCAAGAAAAACATGCTGGT | 225 | 195,536–195,555 |  |  |
|  | CT1291R | AGGATATTTCCCTCAGTTATTCG |  | 195,760–195,738 |  |  |
|  | PCTM3 | TCCTTGCAAGCTCTGCCTGTGGGGAATCCT | 1,019 | 779,977–780,006 | (27) |  |
|  | NR1 | CCGCAAGATTTTCTAGATTTC |  | 779,008–778,988 |  |  |
|  | CT1335F* | AAAGCGTCCTCTGGAAGGG | 208 | 737,198- 737,216 | (28) |  |
|  | CT1335R* | CCTTCTCCTAACAACTTACGC |  | 737,405- 737,385 |  |  |
|  | CT1299F* | ATCGCTTAAGATTCTCGGAGG | 342 | 291,654- 291,674 |  |  |
|  | CT1299R* | AGGTTCTAGCTGAGCATGGG |  | 291,995- 291,976 |  |  |
|  | CT1291F* | ATATAAAAAGAACCGTTGTTTCTG | 329 | 195,462- 195,485 |  |  |
|  | CT1291R* | CATCTTAGACATGCTCCGGC |  | 195,790- 195,771 |  |  |
|  |  |  |  |  |  |  |

^a^ According to D/UW-3/CX, Genbank accession number NC_000117
