# Supplementary material for: High-resolution genotyping of Lymphogranuloma Venereum (LGV) strains of Chlamydia trachomatis in London using multi-locus VNTR analysis-ompA genotyping (MLVA-ompA)
Source: PLoS One. 2021 Jul 8;16(7):e0254233. doi: 10.1371/journal.pone.0254233 (PMC8266103; doi:10.1371/journal.pone.0254233)
Supplement: S3 Table — VNTR type codes of extracts that were assigned non-LGV ompA genotypes (n = 9) or that could not be assigned an ompA genotype (n = 57), were excluded from this table. (DOCX) [file pone.0254233.s003.docx]

**S3 Table.** **VNTR sequence analysis of extracts assigned LGV *ompA* genotypes (n=164).** VNTR type codes of extracts that were assigned non-LGV *ompA* genotypes (n=9) or that could not be assigned an *ompA* genotype (n=57), were excluded from this table.

| **VNTR type codes** | **n** | **CT1335 Variants^a^** | **References** |
| --- | --- | --- | --- |
| 1 | 159 | GAAAAAG-**9T8A**-GCTTTTGT | (18) |
| 5 | 3 | GAAAAAG-**11T8A**-GCTTTTGT | (18) |
| Non-typeable | 2 |  |  |
|  |  | **CT1299 Variants^a^** |  |
| 4a | 1 | TTTTTATTCT-**10C**-T3C-ATCAAA | (22) |
| 9 | 161 | TTTTTATTCT-3C2T-**6C**-ATCAAA | (22) |
| Non-typeable | 2 |  |  |
|  |  | **CT1291 Variants^a^** |  |
| 2b | 146 | AAAATAGTCTA-**8C**-TATTG | (22) |
| 3b | 16 | AAAATAGTCTA-**9C**-TATTG | (21) |
| Non-typeable | 2 |  |  |

^a^ Repeating mononucleotide sequences at each VNTR locus are shown in bold

Flanking region variation is underlined.
