# Supplementary material for: High-resolution genotyping of Lymphogranuloma Venereum (LGV) strains of Chlamydia trachomatis in London using multi-locus VNTR analysis-ompA genotyping (MLVA-ompA)
Source: PLoS One. 2021 Jul 8;16(7):e0254233. doi: 10.1371/journal.pone.0254233 (PMC8266103; doi:10.1371/journal.pone.0254233)
Supplement: S1 Fig — The ompA sequence of L2b/UCH-1/proctitis (Genbank accession no. AM884177.1) was used as a reference. Nucleotide numbers are given according to L2b/UCH-1/proctitis. L2b/D refers to the ompA sequence of the Portuguese L2b/D strain (Genbank accession no. MN094864.1). The ompA sequences of L2/434/Bu (Genbank accession no. AM884176.1) and D/UW-3 (Genbank accession no. NC_000117.1) were included in the alignment. Coloured dots indicate bases that matched L2b/UCH-1/proctitis. (DOCX) [file pone.0254233.s004.docx]

10 20 30 40 50 60 70

....|....|....|....|....|....|....|....|....|....|....|....|....|....|

**L2b/UCH-1/proctitis** **ATGAAAAAACTCTTGAAATCGGTATTAGTGTTTGCCGCTTTGAGTTCTGCTTCCTCCTTGCAAGCTCTGC**

**N018**  **----------------------------------------------------------------------**

**N042**  **----------------------------------------------------------------------**

**N126**  **----------------------------------------------------------------------**

**N161**  **----------------------------------------------------------------------**

**N167**  **----------------------------------------------------------------------**

**N168**  **----------------------------------------------------------------------**

**N175**  **----------------------------------------------------------------------**

**V031**  **----------------------------------------------------------------------**

**L2b/D**  **......................................................................**

**L2/434/Bu**  **......................................................................**

**D/UW-3/Cx**  **.............................A........................................**

80 90 100 110 120 130 140

....|....|....|....|....|....|....|....|....|....|....|....|....|....|

**L2b/UCH-1/proctitis** **CTGTGGGGAATCCTGCTGAACCAAGCCTTATGATCGACGGAATTCTATGGGAAGGTTTCGGCGGAGATCC**

**N018**  **-----------------------------.........................................**

**N042**  **-----------------------------.........................................**

**N126**  **-----------------------------.........................................**

**N161**  **-----------------------------.........................................**

**N167**  **-----------------------------.........................................**

**N168**  **-----------------------------.........................................**

**N175**  **-----------------------------.........................................**

**V031**  **-----------------------------.........................................**

**L2b/D**  **......................................................................**

**L2/434/Bu**  **......................................................................**

**D/UW-3/Cx**  **..............................................G.......................**

150 160 170 180 190 200 210

....|....|....|....|....|....|....|....|....|....|....|....|....|....|

**L2b/UCH-1/proctitis** **TTGCGATCCTTGCACCACTTGGTGTGACGCTATCAGCATGCGTATGGGTTACTATGGTGACTTTGTTTTC**

**N018**  **......................................................................**

**N042**  **......................................................................**

**N126**  **......................................................................**

**N161**  **......................................................................**

**N167**  **......................................................................**

**N168**  **......................................................................**

**N175**  **......................................................................**

**V031**  **......................................................................**

**L2b/D**  **......................................................................**

**L2/434/Bu**  **......................................................................**

**D/UW-3/Cx**  **.............G.............................G.T........C..A............**

220 230 240 250 260 270 280

....|....|....|....|....|....|....|....|....|....|....|....|....|....|

**L2b/UCH-1/proctitis** **GACCGTGTTTTGCAAACAGATGTGAATAAAGAATTCCAAATGGGTGCCAAGCCTACAACTGCTACAGGCA**

**N018**  **......................................................................**

**N042**  **......................................................................**

**N126**  **......................................................................**

**N161**  **......................................................................**

**N167**  **......................................................................**

**N168**  **......................................................................**

**N175**  **......................................................................**

**V031**  **......................................................................**

**L2b/D**  **......................................................................**

**L2/434/Bu**  **......................................................................**

**D/UW-3/Cx**  **............A....T.................T..G......................A........**

290 300 310 320 330 340 350

....|....|....|....|....|....|....|....|....|....|....|....|....|....|

**L2b/UCH-1/proctitis** **ATGCTGCAGCTCCATCCACTTGTACAGCAAGAGAGAATCCTGCTTACGGCCGACATATGCAGGATGCTGA**

**N018**  **......................................................................**

**N042**  **......................................................................**

**N126**  **......................................................................**

**N161**  **......................................................................**

**N167**  **......................................................................**

**N168**  **......................................................................**

**N175**  **......................................................................**

**V031**  **......................................................................**

**L2b/D**  **......................................................................**

**L2/434/Bu**  **......................................................................**

**D/UW-3/Cx**  **..AG................CT................................................**

360 370 380 390 400 410 420

....|....|....|....|....|....|....|....|....|....|....|....|....|....|

**L2b/UCH-1/proctitis** **GATGTTTACAAATGCTGCTTACATGGCATTGAATATTTGGGATCGTTTTGATGTATTCTGTACATTAGGA**

**N018**  **...............C....G.................................................**

**N042**  **...............C....G.................................................**

**N126**  **...............C....G.................................................**

**N161**  **...............C....G.................................................**

**N167**  **...............C....G.................................................**

**N168**  **...............C....G.................................................**

**N175**  **...............C....G.................................................**

**V031**  **...............C....G.................................................**

**L2b/D**  **...............C....G.................................................**

**L2/434/Bu**  **......................................................................**

**D/UW-3/Cx**  **...............C....G.................................................**

430 440 450 460 470 480 490

....|....|....|....|....|....|....|....|....|....|....|....|....|....|

**L2b/UCH-1/proctitis** **GCCACCAGTGGATATCTTAAAGGAAATTCAGCATCTTTCAACTTAGTTGGGTTATTCGGAGATAGTGAGA**

**N018**  **..........................C..T..T........T........A..G..T.......A...A.**

**N042**  **..........................C..T..T........T........A..G..T.......A...A.**

**N126**  **..........................C..T..T........T........A..G..T.......A...A.**

**N161**  **..........................C..T..T........T........A..G..T.......A...A.**

**N167**  **..........................C..T..T........T........A..G..T.......A...A.**

**N168**  **..........................C..T..T........T........A..G..T.......A...A.**

**N175**  **..........................C..T..T........T........A..G..T.......A...A.**

**V031**  **..........................C..T..T........T........A..G..T.......A...A.**

**L2b/D**  **..........................C..T..T........T........A..G..T.......A...A.**

**L2/434/Bu**  **................................................................A.....**

**D/UW-3/Cx**  **..........................C..T..T........T........A..G..T.......A...A.**

500 510 520 530 540 550 560

....|....|....|....|....|....|....|....|....|....|....|....|....|....|

**L2b/UCH-1/proctitis** **ACCATGCTACAGTTTCAGATAGTAAGCTTGTACCAAATATGAGCTTAGATCAATCTGTTGTTGAGTTGTA**

**N018**  **.T..AAAA..G..--..A.GC.-G..TC..................T.......................**

**N042**  **.T..AAAA..G..--..A.GC.-G..TC..................T.......................**

**N126**  **.T..AAAA..G..--..A.GC.-G..TC..................T.......................**

**N161**  **.T..AAAA..G..--..A.GC.-G..TC..................T.......................**

**N167**  **.T..AAAA..G..--..A.GC.-G..TC..................T.......................**

**N168**  **.T..AAAA..G..--..A.GC.-G..TC..................T.......................**

**N175**  **.T..AAAA..G..--..A.GC.-G..TC..................T.......................**

**V031**  **.T..AAAA..G..--..A.GC.-G..TC..................T.......................**

**L2b/D**  **.T..AAAA..G..--..A.GC.-G..TC..................T.......................**

**L2/434/Bu**  **......................................................................**

**D/UW-3/Cx**  **.T..AAAA..G..--..A.GC.-G..TC..................T.......................**

570 580 590 600 610 620 630

....|....|....|....|....|....|....|....|....|....|....|....|....|....|

**L2b/UCH-1/proctitis** **TACAGATACTACTTTTGCTTGGAGTGCTGGAGCTCGTGCAGCTTTGTGGGAATGTGGATGCGCGACTTTA**

**N018**  **..................G.....C.TC..C.....C.......................T..A......**

**N042**  **..................G.....C.TC..C.....C.......................T..A......**

**N126**  **..................G.....C.TC..C.....C.......................T..A......**

**N161**  **..................G.....C.TC..C.....C.......................T..A......**

**N167**  **..................G.....C.TC..C.....C.......................T..A......**

**N168**  **..................G.....C.TC..C.....C.......................T..A......**

**N175**  **..................G.....C.TC..C.....C.......................T..A......**

**V031**  **..................G.....C.TC..C.....C.......................T..A......**

**L2b/D**  **..................G.....C.TC..C.....C.......................T..A......**

**L2/434/Bu**  **......................................................................**

**D/UW-3/Cx**  **..................G.....C.TC..C.....C.......................T..A......**

640 650 660 670 680 690 700

....|....|....|....|....|....|....|....|....|....|....|....|....|....|

**L2b/UCH-1/proctitis** **GGCGCTTCTTTCCAATACGCTCAATCCAAGCCTAAAGTCGAAGAATTAAACGTTCTCTGTAACGCAGCTG**

**N018**  **..A.....A........T........T..A........A....................C..T.....A.**

**N042**  **..A.....A........T........T..A........A....................C..T.....A.**

**N126**  **..A.....A........T........T..A........A....................C..T.....A.**

**N161**  **..A.....A........T........T..A........A....................C..T.....A.**

**N167**  **..A.....A........T........T..A........A....................C..T.....A.**

**N168**  **..A.....A........T........T..A........A....................C..T.....A.**

**N175**  **..A.....A........T........T..A........A....................C..T.....A.**

**V031**  **..A.....A........T........T..A........A....................C..T.....A.**

**L2b/D**  **..A.....A........T........T..A........A....................C..T.....A.**

**L2/434/Bu**  **......................................................................**

**D/UW-3/Cx**  **..A.....A........T........T..A........A....................C..T.....A.**

710 720 730 740 750 760 770

....|....|....|....|....|....|....|....|....|....|....|....|....|....|

**L2b/UCH-1/proctitis** **AGTTTACTATCAATAAGCCTAAAGGATATGTAGGGCAAGAATTCCCTCTTGATCTTAAAGCAGGAACAGA**

**N018**  **..........T.....A........G........TA.G..G..T.............C............**

**N042**  **..........T.....A........G........TA.G..G..T.............C............**

**N126**  **..........T.....A........G........TA.G..G..T.............C............**

**N161**  **..........T.....A........G........TA.G..G..T.............C............**

**N167**  **..........T.....A........G........TA.G..G..T.............C............**

**N168**  **..........T.....A........G........TA.G..G..T.............C............**

**N175**  **..........T.....A........G........TA.G..G..T.............C............**

**V031**  **..........T.....A........G........TA.G..G..T.............C............**

**L2b/D**  **..........T.....A........G........TA.G..G..T.............C............**

**L2/434/Bu**  **......................................................................**

**D/UW-3/Cx**  **..........T.....A........G........TA.G..G..T.............C............**

780 790 800 810 820 830 840

....|....|....|....|....|....|....|....|....|....|....|....|....|....|

**L2b/UCH-1/proctitis** **TGGTGTGACAGGAACTAAGGATGCCTCTATTGATTACCATGAATGGCAAGCAAGTTTAGCTCTCTCTTAC**

**N018**  **..C..C................................................................**

**N042**  **..C..C................................................................**

**N126**  **..C..C................................................................**

**N161**  **..C..C................................................................**

**N167**  **..C..C................................................................**

**N168**  **..C..C................................................................**

**N175**  **..C..C................................................................**

**V031**  **..C..C................................................................**

**L2b/D**  **..C..C................................................................**

**L2/434/Bu**  **......................................................................**

**D/UW-3/Cx**  **..C..C................................................................**

850 860 870 880 890 900 910

....|....|....|....|....|....|....|....|....|....|....|....|....|....|

**L2b/UCH-1/proctitis** **AGACTGAATATGTTCACTCCCTACATTGGAGTTAAATGGTCTCGAGCAAGTTTTGATGCAGACACGATTC**

**N018**  **..................................................C........C..T.......**

**N042**  **..................................................C........C..T.......**

**N126**  **..................................................C........C..T.......**

**N161**  **..................................................C........C..T.......**

**N167**  **..................................................C........C..T.......**

**N168**  **..................................................C........C..T.......**

**N175**  **..................................................C........C..T.......**

**V031**  **..................................................C........C..T.......**

**L2b/D**  **..................................................C........C..T.......**

**L2/434/Bu**  **......................................................................**

**D/UW-3/Cx**  **..................................................C........C..T.......**

920 930 940 950 960 970 980

....|....|....|....|....|....|....|....|....|....|....|....|....|....|

**L2b/UCH-1/proctitis** **GTATTGCTCAGCCGAAGTCAGCTACAACTGTCTTTGATGTTACCACTCTGAACCCAACTATTGCTGGAGC**

**N018**  **....A..C.....A..A.........G..A.T......AC......G..T....................**

**N042**  **....A..C.....A..A.........G..A.T......AC......G..T....................**

**N126**  **....A..C.....A..A.........G..A.T......AC......G..T....................**

**N161**  **....A..C.....A..A.........G..A.T......AC......G..T....................**

**N167**  **....A..C.....A..A.........G..A.T......AC......G..T....................**

**N168**  **....A..C.....A..A.........G..A.T......AC......G..T....................**

**N175**  **....A..C.....A..A.........G..A.T......AC......G..T....................**

**V031**  **....A..C.....A..A.........G..A.T......AC......G..T....................**

**L2b/D**  **....A..C.....A..A.........G..A.T......AC......G..T....................**

**L2/434/Bu**  **......................................................................**

**D/UW-3/Cx**  **....A..C.....A..A.........G..A.T......AC......G..T....................**

990 1000 1010 1020 1030 1040 1050

....|....|....|....|....|....|....|....|....|....|....|....|....|....|

**L2b/UCH-1/proctitis** **TGGCGATGTGAAAGCTAGCGCAGAGGGTCAGCTCGGAGATACCATGCAAATCGTTTCCTTGCAATTGAAC**

**N018**  **.............A..G......................C..A...........................**

**N042**  **.............A..G......................C..A...........................**

**N126**  **.............A..G......................C..A...........................**

**N161**  **.............A..G......................C..A...........................**

**N167**  **.............A..G......................C..A...........................**

**N168**  **.............A..G......................C..A...........................**

**N175**  **.............A..G......................C..A...........................**

**V031**  **.............A..G......................C..A...........................**

**L2b/D**  **.............A..G......................C..A...........................**

**L2/434/Bu**  **......................................................................**

**D/UW-3/Cx**  **.............A..G......................C..A...........................**

1060 1070 1080 1090 1100 1110 1120

....|....|....|....|....|....|....|....|....|....|....|....|....|....|

**L2b/UCH-1/proctitis** **AAGATGAAATCTAGAAAATCTTGCGGTATTGCAGTAGGAACAACTATTGTGGATGCAGACAAATACGCAG**

**N018**  **..................----------------------------------------------------**

**N042**  **..................----------------------------------------------------**

**N126**  **..................----------------------------------------------------**

**N161**  **..................----------------------------------------------------**

**N167**  **..................----------------------------------------------------**

**N168**  **..................----------------------------------------------------**

**N175**  **..................----------------------------------------------------**

**V031**  **..................----------------------------------------------------**

**L2b/D**  **......................................................................**

**L2/434/Bu**  **......................................................................**

**D/UW-3/Cx**  **......................................................................**

1130 1140 1150 1160 1170 1180

....|....|....|....|....|....|....|....|....|....|....|....|....|

**L2b/UCH-1/proctitis** **TTACAGTTGAGACTCGCTTGATCGATGAGAGAGCTGCTCACGTAAATGCACAATTCCGCTTCTAA**

**N018**  **-----------------------------------------------------------------**

**N042**  **------------------------------------------------------**

**N126**  **------------------------------------------------------**

**N161**  **------------------------------------------------------**

**N167**  **------------------------------------------------------**

**N168**  **------------------------------------------------------**

**N175**  **------------------------------------------------------**

**V031**  **------------------------------------------------------**

**L2b/D**  **..................................A..............................**

**L2/434/Bu**  **.................................................................**

**D/UW-3/Cx**  **..................................A..............................**
